# Predictors of psychiatric comorbidity in cancer patients at the time of their discharge from the hospital

**DOI:** 10.1007/s00127-021-02138-1

**Published:** 2021-07-25

**Authors:** Julia Roick, Helge Danker, Anette Kersting, Arne Dietrich, Andreas Dietz, Kirsten Papsdorf, Jürgen Meixensberger, Jens-Uwe Stolzenburg, Hubert Wirtz, Susanne Singer

**Affiliations:** 1 Institute of Medical Sociology, Medical Faculty, Martin Luther University Halle-Wittenberg, Magdeburger Straße 8, 06112 Halle (Saale), Germany; 2 Devision of Medical Psychology and Medical Sociology, University Medical Center Leipzig, Philipp-Rosenthal-Straße 55, 04103 Leipzig, Germany; 3 Department of Psychosomatic Medicine and Psychotherapy, University Medical Center Leipzig, Semmelweisstraße 10, 04103 Leipzig, Germany; 4 Department of Visceral-, Transplantation-, Thoracic-, and Vascular Surgery, University Medical Center Leipzig, Liebigstraße 20, 04103 Leipzig, Germany; 5 Department of Otolaryngology, University Medical Center Leipzig, Liebigstraße 10-14, 04103 Leipzig, Germany; 6 Department of Radiation-Oncology, University Medical Center Leipzig, Stephanstraße 9a, 04103 Leipzig, Germany; 7 Department of Neurosurgery, University Medical Center Leipzig, Liebigstraße 20, 04103 Leipzig, Germany; 8 Department of Urology, University Medical Center Leipzig, Liebigstraße 20, 04103 Leipzig, Germany; 9 Department of Pneumology, University Medical Center Leipzig, Liebigstraße 20, 04103 Leipzig, Germany; 10 Institute of Medical Biostatistics, Epidemiology and Informatics (IMBEI), University Medical Centre of Johannes Gutenberg University Mainz, Obere Zahlbacher Straße 69, 55131 Mainz, Germany

**Keywords:** Cancer, Psychiatric comorbidity, Predictors, Longitudinal study, Prospective study

## Abstract

**Purpose:**

A cancer diagnosis can have a substantial impact on one’s mental health. The present study investigated the prevalence and predictors of psychiatric comorbidities in cancer patients at the time of their discharge from the hospital.

**Methods:**

Psychiatric comorbidities were assessed shortly before hospital discharge and half a year after hospitalization using a structured clinical interview (SCID), based on the diagnostic and statistical manual of mental disorders (DSM-IV). Frequencies at both time points were estimated using percentages and corresponding 95% confidence intervals. Predictors of mental disorders were identified using binary logistic regression models.

**Results:**

At time of hospital discharge, 39 out of 334 patients (12%) were diagnosed with a psychiatric comorbidity, and 15 (7%) were diagnosed half a year later. Among the diagnoses, adjustment disorders (3%) were most frequent at the time of hospital release, while major depression (3%) was the most frequent 6 months later. Having a mental disorder was associated with unemployment (odds ratio (OR) 3.4, confidence interval (CI) 1.1–10.9, *p* = 0.04). There was no evidence that school education (OR 2.0, CI 0.4–9.0, *p* = 0.38), higher education (OR 0.7, CI 0.2–2.4, *p* = 0.60), income (OR 1.0, CI 1.0–1.0, *p* = 0.06), tumor stage (OR 1.1, CI 0.4–3.2, *p* = 0.85), type of disease (OR 0.6, CI 0.2–2.1, *p* = 0.47), pain (OR 1.0, CI 1.0–1.0, *p* = 0.15), fatigue (OR 1.0, CI 1.0–1.0, *p* = 0.77), or physical functioning (OR 1.0, CI 1.0–1.0, *p* = 0.54) were related to the presence of a psychiatric comorbidity.

**Conclusions:**

Unemployment was associated with at least a threefold increased risk of mental disorder, which highlights the need for special attention to be given to this subgroup of cancer patients.

## Introduction

Confrontation with a life-threatening illness such as cancer can lead to anxiety, depression, and other mental health problems in many patients [1–3]. Furthermore, the treatment of this disease and health-related restrictions can be an additional burden for patients [4]. In particular, pain and fatigue have been found to be negatively associated with patients’ well-being and their daily lives [5–7]. As a consequence of the multifarious requirements of a cancer patient, one-third of them suffer from a psychiatric comorbid condition [8–10]. Particularly after diagnosis and at the beginning of the treatment, patients are exposed to a high level of psychological burden. Due to the fact that mental health problems are negatively associated with treatment adherence [11, 12] and cancer survival [13, 14], special attention should be given to such problems. However, they are not always recognized by physicians [15]; thus psychiatric comorbidities can lead to untreated side effects in clinical practice [16]. If left untreated, these mental health conditions can have far-reaching consequences not only for the patients, such as by developing a chronic disease [17], but also for the health care system, such as by increasing costs. For example, it has been shown that psychiatric comorbidity is associated with more frequent treatments and longer hospital stays [17–19].

Physical well-being (e.g., functioning, pain), medical (e.g., tumor stage, type of disease), and sociodemographic characteristics (e.g., age, sex) as well as psychosocial factors (e.g., social support) have been shown to predict anxiety and depressive symptoms in cancer patients [20–23]. The results regarding predictors of structured assessed mental disorders are inconclusive thus far.

This inconclusiveness may result from the fact that in studies on comorbidities, data collection is sometimes carried out using screening instruments and is at other times identified through comprehensive clinical interviews. The studies that have used structured clinical interviews to ascertain psychiatric comorbid diseases have shown that prevalence varies between 23 and 53% [3]. In a study with cancer patients of working age, pain (odds ratio (OR) 1.7), fatigue (OR 1.9), and unemployment (OR 2.0) emerged as predictors of comorbid mental health conditions [24]. In another study of acute care, younger age (< 40 years OR 3.1, 40–49 years OR 2.9), trauma (OR 1.6), and low levels of physical function (OR 2.4) emerged as predictors of the presence of a mental disorder, whereas employment status was not predictive [25]. A study based on national registry data found older age to be a risk factor for psychiatric comorbidities [2]. Results on sex differences have also been inconsistent. While in some studies, comorbidities were higher in female cancer patients [2, 16], others studies did not find any such differences [25, 26].

The aim of the present study is to examine the prevalence of psychiatric comorbidities in cancer patients before their hospital release and half a year later and to investigate potential predictors of comorbid mental health conditions based on the literature shortly before hospital release.

## Methods

### Design and data collection

In this prospective study, all patients admitted to the Visceral, Transplantation, Thoracic, and Vascular Surgery Departments, as well as the Otolaryngology, Radiation–Oncology, Neurosurgery, Urology, and Pneumology Departments of the Leipzig University Hospital between October 2012 and June 2014 for the treatment of cancer were eligible. The exclusion criteria were (a) no histologically confirmed malignancy, (b) age < 18 years, (c) dementia or cognitive restrictions, and (d) insufficient German language skills to participate. Study nurses contacted eligible patients about the content, procedure, and aim of the study shortly after their admission. Patients were approached upon hospitalization (t1), shortly before hospital discharge (t2), 3 months after baseline (t3), and 6 months after baseline (t4). Psychiatric comorbidity was assessed at t2 and t4. Written informed consent was obtained by study nurses from all participants prior to data collection. The study received ethical approval from the Institutional Review Board of Leipzig University (#210-12-02072012)..

### Instruments

*Psychiatric comorbidity* was assessed by study nurses at t2 and t4 using a structured clinical interview (SCID-I) based on the diagnostic and statistical manual of mental disorders (DSM-IV). The SCID is a standardized diagnostic instrument for the assessment of mental health conditions [27]. The following syndromes were assessed: major depressive disorder single episode, dysthymic disorder, adjustment disorder, posttraumatic stress disorder, acute stress disorder, social phobia, specific phobia, general anxiety disorder, panic disorder, alcohol abuse, and alcohol dependence. All the interviewers in this study were trained in conducting the SCID before the start of the study. Each potential diagnosis was discussed by the study team, and in case of doubt, no diagnosis was given. Patients with a diagnosed psychiatric comorbidity in this study did not receive any further treatment.

*Fatigue*, *pain*, *and physical functioning* were measured with the Quality of Life Core Questionnaire of the European Organization for Research and Treatment of Cancer (EORTC), the EORTC QLQ-C30 [28]. The scores were calculated according to the EORTC guidelines [29]. The measures were scaled from 0 to 100, whereby higher scores on the physical functional scale and lower scores on the symptom scales (pain and fatigue) indicate better quality of life. Participants responded using a four-point Likert scale ranging from 0 (“not at all”) to 3 (“very much”). Overall, the scales showed good reliability: fatigue (three items, Cronbach’s alpha = 0.79), pain (two items, Cronbach’s alpha = 0.85), and physical functioning (five items, Cronbach’s alpha = 0.85).

*Education* pertained to (a) the patient’s highest level of primary or secondary education completed (college, postcompulsory education, compulsory education) and (b) the patient’s level of higher education completed (none, apprenticeship, higher, university). Both variables were further dichotomized into compulsory and postcompulsory for “low school education” (versus “high”) and no degrees or apprenticeship as “low higher education” (versus “high”).

To ascertain the equivalent *income* of every patient, the net household income (total income minus taxes) was weighted with the number of people living in the household and their age according to OECD (Organization for Economic Co-operation and Development) standards.

A patient’s *employment status* was classified as follows: patients were coded as “not employed” (versus “employed, in training or retired”) if they were not working at least halftime, were not in training, and were not retired.

*Clinical data* were ascertained from the medical records. Tumor stage was classified according to the Union for International Cancer Control [UICC] classification system [30] and was dichotomized with I + II = “low” and III + IV = “high”. If cancer was recurrent, metastatic or secondary, the type of cancer was classified as “advanced cancer” (versus “not advanced”).

### Statistical analyses

Participants and nonparticipants were compared in terms of basic data available (age, sex, and tumor stage at presentation) using Pearson’s chi-squared and Fisher’s exact tests. Equally, patients who took part in an SCID interview were compared to those who declined concerning demographic data (age, sex), socioeconomic variables (income, school education, higher education, unemployment status), and medical information (tumor stage, type of cancer).

Frequencies of psychiatric comorbidities at t2 and t4 were estimated using percentages and corresponding 95% confidence intervals. To test potential predictors of psychiatric comorbidity, binary logistic regression analyses were performed with having a diagnosed comorbidity (yes/no) as the dependent variable. We only analyzed predictors at hospital discharge (t2) because of the small number of patients with psychiatric diagnoses at t4. Putative risk factors were determined a priori based on the literature and were stepwise included in the models. Independent variables that were not measured continuous were dichotomized before the analyses. The models included the following independent variables: age and sex (model 1); school education (high/low), higher education (high/low), income, unemployment status (yes/no) (model 2); tumor stage (high/low), type of disease (advanced/not advanced) (model 3); and pain, fatigue, and physical functioning at t2 (model 4). For these analyzes, an increase of ten observations in the sample size is necessary for each additional independent variable, whereby it should be noted that for categorical variables each factor level must be treated as one independent variable [31]. Hence, for our analyses, a minimum of 170 observations is necessary which we have fulfilled with our sample size of *N* = 254 (model 4). Data analyses were performed using IBM SPSS® Statistics Version 25.

## Results

### Nonresponder analysis

There was no evidence of differences in age (*p* = 0.44) or sex (*p* = 0.21) between participants and nonparticipants, but there were indications of differences in tumor stage at presentation (*M*_Participants_ = 3.0, *M*_Nonparticipants_ = 3.2 *p* = 0.06).

### Sample characteristics

**Fig. 1:**
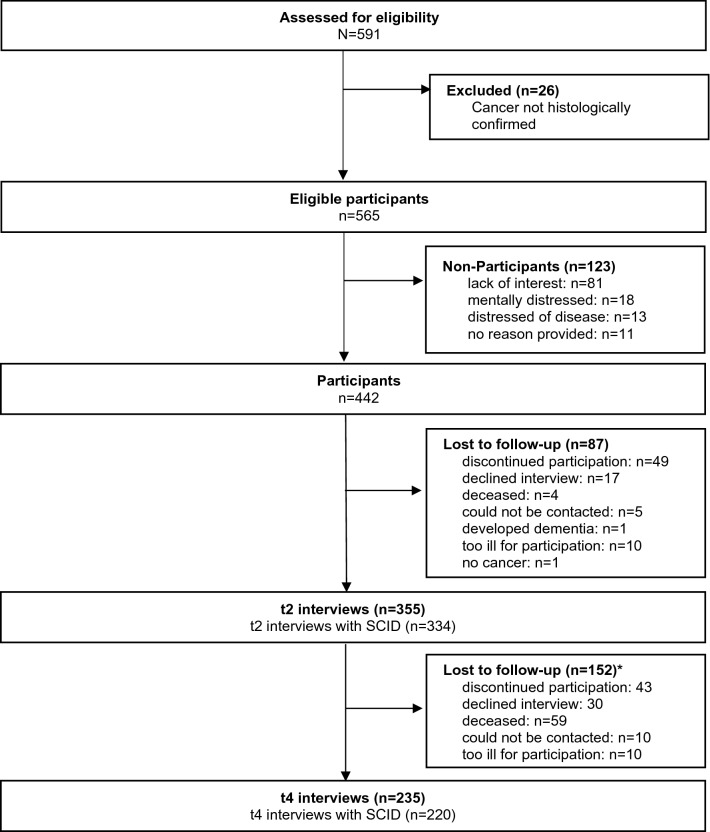
Patient flow through the study. *Including patients who declined or could not be contacted before t2 but participated again later

A total of 591 patients were admitted to the hospital during the study period. Twenty-six patients (4%) had to be excluded because cancer was not histologically confirmed, thereby leaving 565 patients eligible for the study. Of those patients, 123 (22%) declined study participation, resulting in 442 participants (Fig. 1). The most frequent tumor sites were head and neck (19%), prostate (18%), urinary organs (11%), brain (9%), lung (8%), and colorectal (7%). The mean age of the participants was 64 years (Table 1). The majority of the participants were male (70%), had advanced cancer (64%), and were either employed, retired or in training (70%).

**Table 1:** Demographic and medical characteristics of the study participants (*N* = 442)

Variables	Number	Percent
Age at baseline		
Mean [SD] in years	63.6 [11.0]	
Sex		
Female	132	29.9
Tumor stage		
0/I	50	11.3
II	90	20.4
III	92	20.8
IV	191	43.2
Unknown	19	4.3
Type of cancer		
Advanced	283	64.0
Not advanced	140	31.7
Unknown	19	4.3
Cancer site		
Head and neck	83	18.8
Prostate	81	18.3
Urinary organs	47	10.6
Brain	38	8.6
Lung	36	8.1
Colorectal	31	7.0
Other	126	28.6
Equivalence income		
< 500 euros	18	4.1
500 to 999 euros	127	28.7
1000–1499 euros	83	18.8
> 1500 euros	122	27.6
Unknown	92	20.8
School education		
Compulsory	108	24.4
Post-compulsory	245	55.4
High school	86	19.5
Unknown	3	0.7
Vocational training		
None	19	4.3
Apprenticeship	232	52.5
Higher	77	17.4
University	111	25.1
Unknown	3	0.7
Employment status		
Not employed	131	29.6
Employed, retired, or in training	308	69.7
Unknown	3	0.7

Of the 442 participants, 355 (80%) participated in the study again at hospital discharge (t2), and of these, 334 (94%) agreed to take part in the SCID interview. Half a year after baseline (t4), 235 patients (53%) were interviewed, and 220 (94%) agreed to participate in an SCID interview. Participants with and without an SCID interview at t2 did not differ in age (*p* = 0.46), sex (*p* = 0.14), tumor stage at diagnosis (*p* = 0.39), type of cancer (*p* = 0.81), unemployment status (*p* = 0.09), school education (*p* = 0.56), or higher education (*p* = 0.62). However, there was evidence of differences in income (*p* = 0.03) between participants with and without an SCID interview; those with low income declined to take part more often.

### Frequency of psychiatric comorbidity

At t2, 39 patients (12%) were diagnosed with a psychiatric comorbidity according to the SCID interview (Table 2). The most common comorbid diseases were adjustment disorder (*n* = 10, 3%), major depression (*n* = 8, 2%), and alcohol dependence (*n* = 7, 2%). One participant (0.3%) had two diagnoses: major depression and specific phobia. 6 months after baseline, 15 patients (7%) were diagnosed with at least one psychiatric disorder. Similar to t2, major depression (*n* = 7, 3%), specific phobia (*n* = 4, 2%), and alcohol dependence (*n* = 3, 1%) were the most common diagnoses. Two patients (1%) had a dual diagnosis: major depression and panic disorder, and major depression and specific phobia.

**Table 2:** Frequencies and 95% confidence intervals of mental disorders

Mental disorder^a^	t2 (*N* = 334)			t4 (*N* = 220)		
	*N*	%	95% CI	*N*	%	95% CI
Major depression	8	2.4	1.1–4.5	7	3.2	1.4–6.1
Dysthymic disorder	1	0.3	0–1.4	0	0	–
Social phobia	0	0	–	0	0	–
Specific phobia	5	1.5	0.6–3.2	4	1.8	0.6–4.3
Panic disorder	0	0	–	1	0.5	0–2.1
Generalized anxiety disorder	2	0.6	0.1–1.9	0	0	–
Posttraumatic stress disorder	1	0.3	0–1.4	0	0	–
Acute stress disorder	3	0.9	0.3–2.4	0	0	–
Adjustment disorder	10	3.0	1.5–5.2	2	0.9	0.2–2.9
Alcohol abuse	3	0.9	0.3–2.4	0	0	–
Alcohol dependence	7	2.1	0.9–4.1	3	1.4	0.4–3.6
Any disorder	39	11.7	8.6–15.4	15	6.8	4–10.7

t2 = hospital discharge; t4 = 6 months after baseline

^a^Dual diagnosis possible

### Predictors of psychiatric comorbidity

In the multivariate model, we found that when controlling for other socioeconomic and medical variables, unemployed patients had 3.4-fold higher odds of having a psychiatric comorbidity (95% CI 1.1–10.9, *p* = 0.04) at t2 than patients with full or halftime employment, patients who were in training, or pensioners (Table 3). Other socioeconomic variables (school, higher education and income) and medical characteristics, as well as fatigue, pain, and physical functioning, showed no association with psychiatric comorbidities in our study.

**Table 3:** Predictors of psychiatric comorbidity at hospital discharge

Predictor	Reference	Model 1 (*N* = 334)^a^			Model 2 (*N* = 271)^a^			Model 3 (N = 257)^a^			Model 4 (N = 254)^a^		
		OR	CI	*p*	OR	CI	*p*	OR	CI	*p*	OR	CI	*p*
Sex	Male	1.8	0.9–3.5	0.11	1.9	0.9–4.2	0.11	1.9	0.8–4.6	0.15	2.0	0.8–5.3	0.15
Age	< 63 years	0.7	0.4–1.4	0.30	1.0	0.4–2.7	0.99	1.0	0.3–2.8	0.99	1.3	0.4–3.9	0.70
School education	High				1.1	0.3–4.0	0.84	1.5	0.4–5.8	0.55	2.0	0.4–9.0	0.38
Higher education	High				0.7	0.3–2.2	0.59	0.6	0.2–1.9	0.43	0.7	0.2–2.4	0.60
Income					1.0	1.0–1.0	0.06	1.0	1.0–1.0	0.08	1.0	1.0–1.0	0.06
Employment status	Employed, retired or in training				2.7	1.0–7.6	0.05	3.1	1.1–9.2	0.04	3.4	1.1–10.9	0.04
UICC	I/II							1.0	0.4–2.8	0.95	1.1	0.4–3.2	0.85
Type of disease	Not advanced							0.6	0.2–1.8	0.37	0.6	0.2–2.1	0.47
Pain at t2											1.0	1.0–1.0	0.15
Fatigue at t2											1.0	1.0–1.0	0.77
Physical functioning at t2											1.0	1.0–1.0	0.54

OR = odds ratio; CI = 95% confidence interval

Model 1: includes age and sex as predictors; Model 2: + socioeconomic variables, Model 3: + medical variables, Model 4: + pain, fatigue, and physical functioning

^a^Different sample sizes because of missing data in predictor variables

## Discussion

The present study investigated the prevalence of comorbid psychiatric diagnosis according to SCID shortly before hospital discharge and half a year later in cancer patients.

Our results show that 12% of the patients suffered from a psychiatric comorbidity at time of hospital release, and 7% suffered from a psychiatric comorbidity half a year after their hospital stay. Compared to other studies that have reported a prevalence of 30% in acute care, the proportion in this study is considerably lower [8, 32]. This discrepancy may be explained by the fact that we did not include the consumption of nicotine and drug dependence as mental health conditions, although they were classified as one in the SCID interview. Furthermore, in our study, older patients (> 70 years) were more frequently included than in other studies (30% versus 20%) [25, 32]. Other studies have found that younger cancer patients are more likely to suffer from psychiatric comorbidities [25, 33]. Another reason for this difference could be that the abovementioned studies about mental disorders in acute care conducted their clinical interviews at the beginning of the patient’s hospital stay, while we did so at the time of hospital discharge. Furthermore, not all patients had just received their diagnosis; in some cases, the initial diagnosis had already occurred years ago.

In our study, the most frequent category of psychiatric comorbidity after hospitalization was adjustment disorder, followed by major depression. Half a year after baseline, major depression followed by a specific phobia were the most common. This result is similar to other studies that have found these disorders to be the most common [1, 24, 32].

Another aim of the study was to investigate predictors of psychiatric comorbidity shortly before hospital discharge. We found that unemployed patients were three times more likely to have a diagnosis according to the SCID compared to full- or halftime employed patients, persons in training, and pensioners. This result is in line with previous studies showing that cancer patients who are unemployed or those who had to suspend their work due to their disease suffer more frequently from comorbid psychiatric disorders [24, 34]. The number of mental disorders is also higher in unemployed persons in the general population [35]. The more common comorbidities in unemployed individuals may be because the loss of the job leads to a decrease in one’s social network and thus to reduced social support, which has been found to negatively influence the development of psychological comorbidities in cancer patients [36].

Clinicians should have a special focus on unemployed patients because they seem to be a specific at-risk group who may need additional support. Screening instruments could help in an economical way to identify if an unemployed patient is in need of health care. Referral to cancer support services or psycho-oncological treatment seems to be of great importance, especially because it has been shown that many cancer patients express a desire for psychosocial help [32]. In the case of patients with psychiatric comorbid conditions, clinicians should also point out the possibility of psychotherapeutic support since there is an undersupply of mental health care in cancer patients [24]. In addition, clinicians should discuss with their patients work-related issues and the idea of returning to work because these topics are often neglected [37]. If it then becomes apparent that support is necessary, the patients should be referred to occupational health professionals. However, there are also studies that report no association between employment status and psychiatric diagnoses in cancer patients [33, 38]. All the other predictors examined in this study showed no association with psychiatric comorbidities. However, this outcome could also have occurred because of the small sample size.

The study has several limitations that need to be considered when interpreting the results. First, we found psychiatric comorbidities much less often than in other studies with cancer patients; therefore, we do not know if our study sample is representative of the whole population. Another explanation may be that the prevalence of mental disorders varies between countries due to differences in the organization and financing of health care and social security systems. Since the German health care system offers comprehensive insurance coverage and a high level of health services, the lower number of psychiatric comorbidities in our study could be due to this fact. Second, we had no information about the psychiatric comorbidities of the patients before their cancer diagnosis, which could have been an important predictor as well. Third, we have to note that our results about associations between unemployment and mental disorders may be biased by the large differences in group size between employed and unemployed patients. Finally, because of the small number of mental disorders, we had to limit our analyses to a few predictors. We also did not examine predictors of psychiatric comorbidity half a year after hospitalization because of the low prevalence of mental disorders. Even with these limitations, the current study provides insight into predictors of psychiatric comorbidities in cancer patients who were assessed with structured clinical interviews at the time of their discharge from the hospital.

In summary, our study has shown that one in ten patients suffer from a psychiatric comorbidity at the end of their hospitalization. Unemployed patients should be given special attention because they are three times more likely to suffer from a mental disorder than other cancer patients. For these patients, it might be helpful for them to discuss their problems with hospital social services or to be referred to ambulant cancer care services.

## Data Availability

The dataset analyzed in the current study is not available publicly as eligible patients were informed at the time of the survey their data would be stored securely and confidentially. The data may be available from the corresponding author upon reasonable request.
